# A tangible prospect for the treatment of gingivitis using a potentially probiotic strain *Lactobacillus plantarum* MK06 isolated from traditional dairy products: a triple blind randomized clinical trial

**DOI:** 10.1186/s12903-023-03494-x

**Published:** 2023-11-16

**Authors:** Sima Modiri, Mohadeseh Heidari, Rojin Shahmohammadi, Leila Jabbareh, Avideh Maboudi, Mahmood Moosazadeh, Hojatollah Vali, Kambiz Akbari Noghabi

**Affiliations:** 1 National Institute of Genetic Engineering and Biotechnology (NIGEB), P. O. Box 14155-6343, Tehran, Iran; 2 Department of Periodontology, Faculty of Dentistry, Mazandaran University of Medical Sciences, Sari, Iran; 3 Present Address: Implant Research Center, Dental Research Institute, Tehran University of Medical Sciences, Tehran, Iran; 4 Department of Periodontics, School of Dentistry, Tehran University of Medical Sciences, Tehran, Iran; 5 Department of Periodontology, Faculty of Dentistry, Dental Research Center, Mazandaran University of Medical Sciences, Sari, Iran; 6 Gastrointestinal Cancer Research Center, Mazandaran University of Medical Sciences, Sari, Iran; 7 Department of Anatomy & Cell Biology, McGill University, 3640 Street, Montreal, QC H3A 0C7 Canada

**Keywords:** *Lactobacillus plantarum* MK06, Dairy products, Probiotics, Mouthwashes, Gingivitis, Oral health, Gingival index, Dental plaque index

## Abstract

**Background:**

Frequent bacterial plaque buildup at the gingival margin and crevice can provoke an inflammatory reaction in gingival tissues which manifests as gingivitis. Probiotics could serve as a beneficial complementary therapy for treating gingival inflammation. The main aim of this research was to investigate the effect of the *Lactobacillus plantarum* MK06 probiotic strain on the treatment of gingivitis.

**Methods:**

Patients with gingivitis, who were referred to a private clinic and were systematically healthy, were included in this randomized, triple-blind, placebo-controlled trial. They were instructed to use either placebo or *Lactobacillus plantarum* suspensions for one minute two times a day after tooth-brushing for four weeks. Then, the clinical parameters of gingivitis, including plaque index (PI), gingival index (GI), bleeding on probing (BOP), and oral hygiene index (OHI-s), were measured in the first, second, and fourth weeks. A total of forty-two patients were randomly assigned to the experimental (n = 21) and control (n = 21) groups. The mean age of the experimental and control groups was 29.10 and 28.48, respectively.

**Results:**

The mean scores of BOP, GI, PI, and OHI-s reduced over time in both the control and test groups. However, according to the Mann-Whitney test, the difference between the two groups was not significant at the same time intervals (P ≥ 0.05) and only GI showed a significant difference in the fourth week (GI-3, P = 0.006). Nevertheless, the experimental group experienced a higher overall reduction rate than the control group. The BOP, GI, PI, and OHI-s scores decreased by 0.081, 0.204, 0.186, and 0.172 times in the second week, respectively, resulting from the interaction of time and the intervention, which considerably diminished these indices.

**Conclusion:**

This study shows the potential of the probiotic *Lactobacillus plantarum* MK06 suspension as a promoting therapeutic adjuvant in the treatment of gingivitis.

## Introduction

According to the world health organization, oral health is crucial to overall wellness and quality of life. It is described as a state of being without any mouth and facial pain, oral diseases such as tooth cavity, gum disease, tooth loss, oral cancer, and other disorders which can limit a person’s normal activity of chewing, smiling, speaking, and social life [1]. The oral microbiota, the diverse microbial population found in the human mouth cavity, can cause inflammatory conditions, including gingivitis and peri-implant mucositis when its balance is disturbed. As a result, probiotic therapy and oral microbiota replacement therapy have received much attention in recent years as methods of controlling periodontal disease [1].

Periodontal diseases including gingivitis and periodontitis are among prevalent oral diseases in the general population [2]. Gingivitis is classified in two categories, dental biofilm induced gingivitis and non-dental biofilm induced gingivitis. Dental biofilm induced gingivitis is an inflammatory lesion contained to gingiva without extension to periodontal attachment apparatus. It can be associated with dental biofilms alone, mediated by systemic or local risk factors and drug influenced gingival enlargement. It can occur on intact periodontium or reduced healthy periodontium. It is a reversible condition, so treatment modalities such as antiseptic treatment, mechanical debridement, drug changes, or modification in local risk factors can be used [2, 3].

Frequent bacterial plaque buildup at the gingival margin and crevice can provoke an inflammatory reaction in gingival tissues which manifests as gingivitis with the main signs of bleeding, swelling, and redness of gums [3, 4]. If left untreated, this condition may proceed to an advanced and destructive disease known as periodontitis [5]. Therefore, treating gingivitis is a primarily preventive approach [6].

Mechanical removal of dental biofilm through professional scaling and root planning together with regular at-home oral hygiene practice significantly reduces periopathogens [7]. Although mechanical instrumentation is highly effective, microbial recolonization is unpredictable. Also, many people still do not follow oral hygiene guidelines effectively. Therefore, the use of adjunctive antimicrobial agents as chemical plaque control is advised in patients who are more susceptible to gingivitis [8]. However, these chemicals may have adverse effects especially in long-term use [9]. This indicates the need for a safer alternative measure. Recently, there has been increased interest in probiotics, defined as sufficient numbers of live microorganisms that can confer health benefits to the host [10]. They can manipulate the oral microflora by strengthening the resident bacteria and preventing the adhesion and colonization of pathogens [11]. Several studies have shown positive results concerning reduced plaque accumulation and gingival indices following probiotic treatment [12]. Some studies show that using probiotics can modulate the host’s immune response [13, 14]. The effects of different probiotic strains of *Bifidobacterium* and *Lactobacillus plantarum* on oral health have previously been evaluated [15–18]. However, the clinical influences of these probiotic strains regarding gingival inflammation have not been discussed. In a systematic review, the role of probiotics on experimental gingivitis was examined and discussed in detail. A positive effect was observed in which the reduction of gingival crevicular fluid occurred after taking probiotics [19]. Another systematic review and meta-analysis were conducted to address the clinical effectiveness of probiotics in treating gingivitis. After analyzing the results obtained from clinical trials, limited evidence was presented to show the beneficial effects of probiotics in reducing inflammatory parameters due to the high heterogeneity [20]. *Lactobacillus plantarum* strain MK06 is a native probiotic bacterium isolated from traditional dairy products in our previous study [21]. Given that this probiotic bacterial strain prevents the growth of *streptococcus mutans* (the causal agent of tooth decay), its inhibitory effect on gingivitis and periodontal inflammation was investigated. The effect of *Lactobacillus plantarum* suspension on gingivitis-related parameters, including BOP, GI, PI, and OHI, was carefully examined compared to placebo suspension.

## Materials and methods

### Preparation of the probiotic mouthwash

#### Isolation and characterization of the probiotic strain

In order to isolate the *Lactobacillus plantarum* strain, 1 ml of traditionally produced yogurt was dissolved in 0.9% sterile saline and diluted to 1 × 10^− 3^ using a 10-fold serial dilution protocol. 0.1 ml of 10-fold dilution was spread on an MRS agar plate (Merck, Darmstadt, Germany) and incubated for up to 48 h at 30 °C under aerobic conditions. The colonies were stored in MRS broth supplemented with 50% (v/v) glycerol at -70 °C. The isolate was carefully chosen based on its morphological features and catalase examination. Additional analysis was carefully performed to delineate the probiotic characteristics of the isolate as detailed in our previous study [21]. Besides, the 16 S-rRNA gene analysis using universal primers (27 F and 1492R) was carried out to further recognize the bacterial isolate. To this end, the extraction of DNA genomic was done using a DNA extraction kit (SinaClon Co., Tehran, Iran), and the 16 S rRNA gene was amplified through the polymerase chain reaction (PCR) as descrobed before [21]. The partial sequence of the 16 S rRNA gene of this strain has been deposited in the NCBI with accession number of MW314598.1.

#### Probiotic suspension preparation

At this stage, an optimized medium, which was previously developed in our laboratory, was used to culture probiotic bacterium [22]. This culture medium is composed of whey (40 g/L), sodium acetate (5 g/L), peptone (8 g/L), yeast extract (4 g/L), magnesium sulfate (0.2 g/L), ammonium sulfate (2 g/L), Tween 80 (1 g/L), yeast extract (industrial, 4 ml/L). All medium components were purchased from Merck (Darmstadt, Germany). To prepare inoculum, a stock culture of *Lactobacillus plantarum* MK06 was cultivated twice on an MRS agar medium, and a pure colony was inoculated in an optimal liquid culture medium to prepare pre-culture. The culture medium was then incubated under aerobic conditions at 30 °C and 180 rpm. After reaching the exponential growth phase, the bacteria were inoculated in a 500 ml Erlenmeyer flask containing 50 ml of the optimal culture medium so that the initial OD_600_ was 0.1. Bacteria were incubated under the same conditions as pre-culture, and after reaching the mid-exponential growth phase (OD = 2), the cells were isolated by centrifugation at 7000 ×g for 10 min. The collected cells were washed three times with phosphate-buffered saline (PBS) and re-dissolved in the PBS buffer to prepare the cell solution. 1 ml aliquots were prepared from the bacterial solution in a 1.5 ml vial so that each vial contained 10^8^ CFU/ml. The probiotic powder was then prepared by placing the vials in the freeze-drying overnight.

#### Instruction for the probiotic mouthwash

A potential probiotic mouthwash was made as a powder in a 20 ml glass tube containing 10^8^ colony forming units (CFU) ml^− 1^ of the freeze-dried *Lactobacillus plantarum* MK06 blended with 100 mg of food-grade maltodextrin (Foodchem, China) as a bulking agent. The prepared single-dose product containers were stored and sealed at room temperature until use. The subjects were instructed to mix the product with 15 ml of tap water before use. The reconstituted potential probiotic suspension was then swished for 30s before being expectorated [23]. Patients were trained to use probiotic mouthwash two times a day for four weeks immediately after brushing, rinse defined amount (not swallow) for 1 min.

### Clinical trial protocol

#### General information

A summary of the clinical part of this study is provided in this section, and more details of the work protocol are available in the supplementary file. The clinical part of this study was performed in the department of periodontology, faculty of density, Mazandaran University of Medical Sciences under the guidance of Dr. Mohadeseh Heydari. The start and end days of the clinical trial were 2018.09.22 and 2019.09.22, respectively.

The clinical trial protocol has been deposited in the Iranian Registry of Clinical Trial (IRCT) network (07/04/2019, IRCT20151017024573N7), entitled as “Evaluation of the effects of the probiotics suspension containing *Lactobacillus plantarum* on gingivitis”.

#### Study goals

##### Primary objective

The aim was to investigate the effect of probiotic bacterium *Lactobacillus plantarum* MK06 on gingivitis.

##### Secondary objectives

The clinical inflammatory parameters including PI, GI, BOP, and OHI-s were evaluated and compared as index parameters related to gingivitis in placebo (control) and experimental groups.

#### Study design

##### Type of study

This study is randomized, triple-blind, placebo-controlled clinical trial with a parallel group design of 42 patients, whose demographic information as well as their clinical parameters had been recorded (Table 1).

**Table 1:** Demographics and diagnostic information (n = 42)

Sex	Male Female	
Mean age	Experimental group Control group	29.10 28.48
Inclusion criteria	Functional teeth Plaque-induced gingivitis	20 functional teeth (5 in each quadrant) GI ≥ 1
**Diagnosis**	Plaque index (PI) Gingival index (GI) Bleeding on probing (BOP) Oral hygiene index (OHI-s)	

##### Research population

Forty-two patients were chosen based on eligibility criteria as follows:

- Criteria for inclusion.

The participants (patients), who meet the inclusion criteria for gingivitis, were over 18 years of age, having at least twenty functional teeth (five in each quadrant) and plaque-induced gingivitis GI ≥ 1 [24].

- Criteria for exclusion.

The persons with the following conditions: Probing depth > 3 mm, any systemic disease or condition capable of modifying the gingival tissues (pregnancy or breastfeeding, diabetes, immune system disorders), history of taking antibiotics in the past three months, taking medications that can affect gingival tissues (steroidal and non-steroidal anti-inflammatory drugs, calcium channel blockers, phenytoin, cyclosporine A), history of allergy, undergoing periodontal treatment during the last six months, active carious lesions, undergoing orthodontic treatment, having crowns or implants, and mouth breathing excluded from the study.

#### Methodology

##### Interventions

In this study, the control group trained to use the dextrose mouthwash, and the intervention group used *Lactobacillus plantarum* probiotic mouthwash. It was also recommended not to use other probiotic products during the study period.

##### Randomization and group allocation

Block randomization, which was used to divide the participants into two groups, was performed in six blocks of seven and assignment made with random allocation software (RAS). The test and control groups were allocated as follows: group (a) Twenty-one individuals assigned to use the *Lactobacillus plantarum* MK06 suspension; group (b) Twenty-one individuals assigned to use a placebo suspension with the same color and appearance. For allocation concealment, forty-two sealed non-transparent envelopes were prepared and opened right before the interventions by principal investigator. During the whole study, both clinicians and participants, and research staff were completely unaware of group assignment.

##### Procedures

**Fig. 1:**
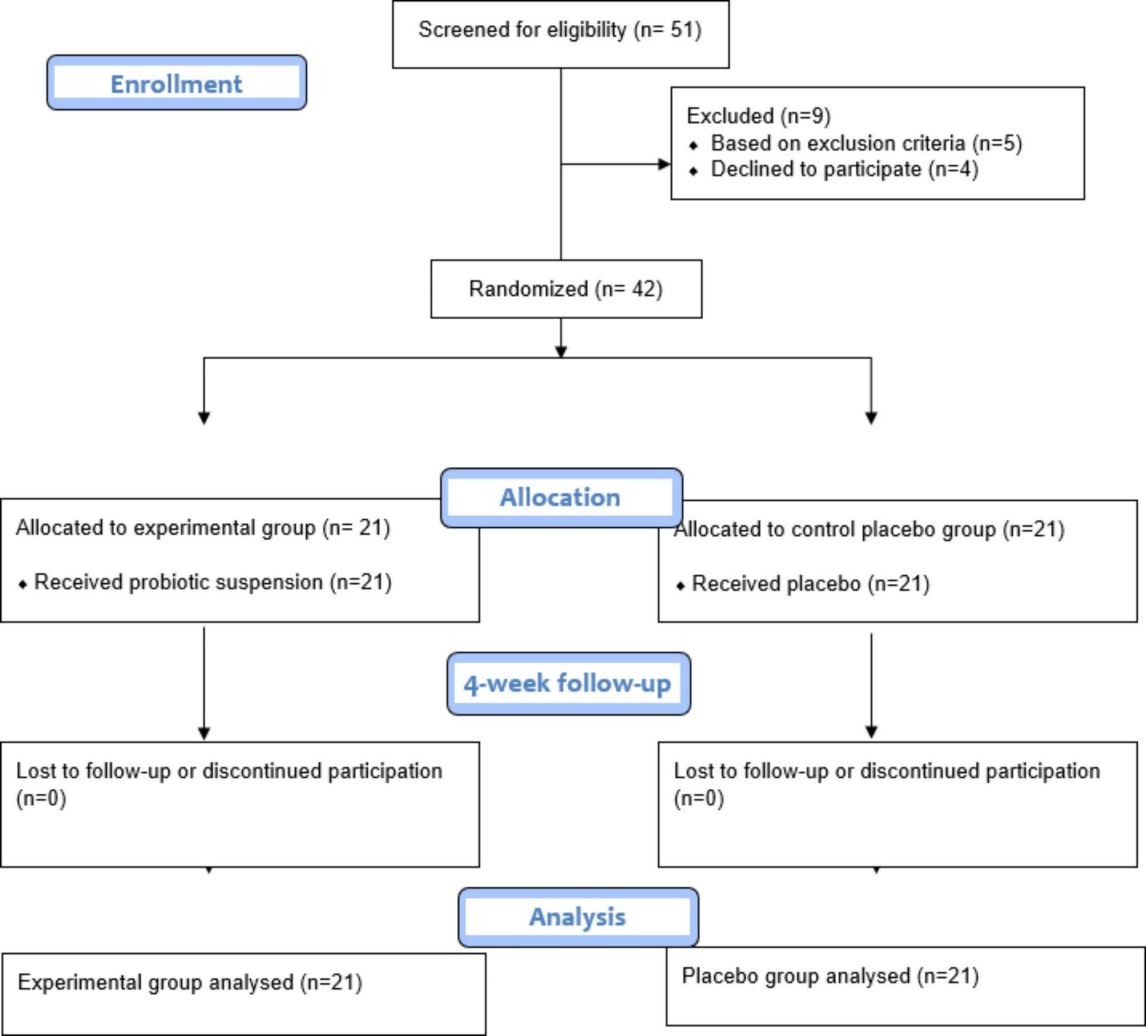
The flow diagram depicts the different stages of research

A potential probiotic suspension of *Lactobacillus plantarum* MK06 and placebo suspension were administered in experimental and control groups, respectively (all suspensions were prepared at the National Institute of Genetic Engineering and Biotechnology). Patients in both groups were instructed to take the suspension two times a day for four weeks, immediately after brushing. At the end of the first, second, and fourth weeks, PI, GI, OHI-s, and BOP indices were evaluated and recorded, based on the criteria mentioned in Tables 2, 3 and 4, and 5 [25–27]. A graphic outline of the study design and procedures has been illustrated in Fig. 1.

**Table 2:** Diagnostic criteria for the Loe & Silness (1963) gingival index (GI)

Score	Criteria
0	Normal gingiva
1	Mild inflammation- slight change in color, mild edema, no bleeding upon probing
2	Moderate inflammation- redness, glazing and swelling, bleeding upon probing
3	Severe inflammation- noticeable redness and edema, tendency to spontaneous bleeding.

**Table 3:** Diagnostic criteria for the Loe & Silness (1963) plaque index (PI)

Score	Criteria
0	No plaque
1	Little plaque accumulation at the gingival margin that can only be seen by moving a probe at the marginal surface
2	Moderate plaque accumulation at the gingival margin that is visible
3	Considerable amount of plaque accumulation on the tooth surface

**Table 4:** Diagnostic criteria for bleeding on probing (BOP) developed by Mühlemann (1977)

Score	Criteria
0	No bleeding
1	Only one bleeding spot
2	Several isolated bleeding spots
3	Interdental papilla triangle filled with blood
4	Extensive bleeding that spreads over the gingival margin

**Table 5:** Diagnostic criteria for oral hygiene index (OHI-s) described by Greene & Vermillion (1964)

Score	Criteria
0	No debris detectable
1	Soft debris that does not cover more than one third of the tooth surface or existence of stains without any debris, regardless of the covered surfaces
2	Debris covering more than one third but less than two thirds of the tooth surface
3	Soft debris covering more than two thirds of the tooth surface

##### Measurement of gingivitis

The four indexes including plaque index (PI), gingival index (GI) [28], presence/absence of bleeding on probing (BOP) [4], and oral hygiene index (OHI-s) are commonly used in recent studies to evaluate gingivitis.

In this study, at baseline (Day 0), forty-two potential patients were examined by a single clinician. Indeed, at the beginning (Day 0), clinical parameters of gingivitis were measured by a single calibrated clinician who was blinded to group assignment. In both groups, the mean score of all BOP, GI, PI, and OHI-s parameters were measured and compared over time to investigate the effects of probiotic *Lactobacillus plantarum* MK06 in the treatment of gingivitis.

- Gingival index (GI).

One of the significant indexes influential in the condition of the gums is the gingival index, which has four levels. Each tooth is divided into four levels, and each one is given 3 points according to the table. If the total score of each level is being divided by four, a gingival index is obtained. The diagnostic criteria for GI employed in this study are described by Loe & Silness (1963) (Table 2).

- Plaque index (PI).

The diagnostic criteria used for the PI, based on Loe & Silness (1963), are given in Table 3.

- Presence/absence of bleeding on probing (BOP).

Bleeding gums is caused by probe stimulation, indicating the progression of the disease. BOP was assessed upon probing at four sites (mesio-buccal, mid-buccal, disto-buccal, and mid-lingua) and sorted according to the description by Mühlemann (1977) (Table 4).

- Oral hygiene index (OHI-s).

An index for measuring oral health as demonstrated according to the assortment in Table 5 [27].

#### Follow-up

The outcome measures of the clinical parameters associated with gingivitis were documented at baseline, first, second and fourth weeks for each group.

#### Data management and statistical analysis

##### Sample size

The calculation was based on the BOP index of Slawik’s study, and its mean values were 76.23 ± 9.11 and 25.8 ± 72.12 for the test and control groups at day 14, respectively. The sample size of the mentioned indicator, with 95% confidence level and 90% test power for two test ranges, was estimated as equal to 30 participants (15 in each experimental and control group) using the formula of comparison between means in G-power software. It should be noted that considering the dropout rate of 40%, the final sample volume has been increased to forty-two (twenty-one in each group).

##### Statistical analysis of the data

The collected data were entered into SPSS 22 software, and quantitative and qualitative variables were reported using mean ± standard deviation and descriptive statistics, respectively. The normality of the associated variables was examined using the Kolmogorov-Smirnov test. Descriptive Properties of the variables were presented using frequency, mean and standard deviation. Mean comparison of indices between intervention and control groups, at each stage of measurement, was performed by independent t-test or its non-parametric equivalent, Mann-Whitney. Mean comparison before and after in each group was performed by paired t-test or its non-parametric equivalent statistical test, Wilcoxon. Also, the comparison of the mean changes of scores overtime was performed by generalized estimator equations (GEE) test, repeated measures analysis of variance, or its non-parametric equivalent, Friedman test.

### Ethics

This protocol was approved by the ethics committee of Mazandaran University of Medical Sciences, Iran (IR.MAZUMS.REC.1396.2950). This study was performed and reported following the Consolidated Standards of Reporting Trials (CONSORT) Statement [28]. All eligible patients, who met the criteria of interest, were given the necessary knowledge of the experiment details. Informed consent was obtained from all participants. All experiments were performed in accordance with relevant guidelines and regulations.

### Conflict of interest

Authors declare no conflict of interest regarding this trial.

### Data availability

The datasets generated during and/or analyzed during the current study are not publicly available due to minimal datasets that would be necessary to interpret, but are available from the corresponding author on reasonable request.

## Results

### Participant flow

In this clinical trial study, a total of fifty-one participants were screened, and nine were excluded either for not meeting the inclusion criteria or declined to take part in this study. The remaining forty-two patients were recruited and randomly divided into test and control groups. The study flow diagram following CONSORT guidelines has been shown in Fig. 1.

The mean age was 29.10 and 28.48 in experimental and control groups, respectively, and according to the Mann-Whitney test, there was no significant difference between the two groups (p = 0.940). Moreover, based on the Mann-Whitney test, there was no significant difference between the two groups regarding gender distribution (p = 1.333). In terms of distribution of smoking, the rate was higher in the intervention group than the control group, which was not statistically significant based on the results of Fisher’s test (23.8% Vs 19%; p = 0.707).

### Harms

No adverse effects had been reported by patients based on the questionnaire.

### Subgroup analyses

#### Bleeding on probing (BOP)

**Fig. 2:**
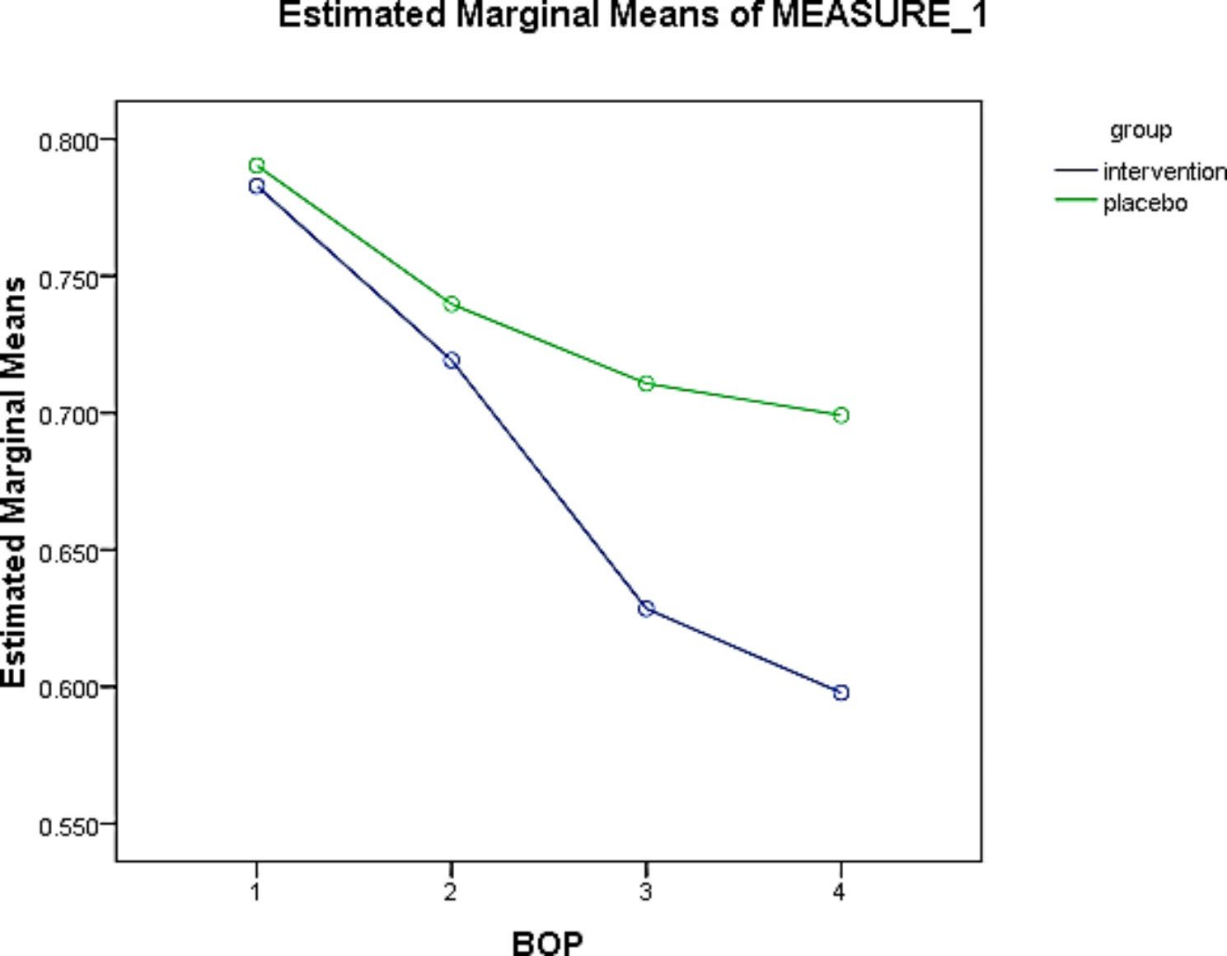
Comparison of the effect of *L. plantarum* probiotic suspension on mean BOP values at 4 weeks

The mean BOP scores at baseline (BOP-0) in the intervention and placebo groups were 0.78 and 0.79, respectively, but this difference was not statistically significant (p = 0.999). The mean BOP at four weeks (BOP-3) were 0.59 and 0.69 in the intervention and placebo groups, respectively, in which this difference was not statistically significant (p = 0.085). According to the results of the Friedman test, during the study time, the changes in BOP score had a statistically significant decreasing trend in both groups (p = 0.001) (Table 6). The percentage of BOP changes was 24.35% in the intervention and 12.65% in the placebo groups, which may be clinically significant. However, there was no statistically significant difference between the two groups. Based on the generalized estimator equations model and considering that the significance of this test is less than 0.05, the time effect was significant (p = 0.001). In other words, the difference between the mean of the measurements in different time periods based on the Wald Chi-Square test was significant (Table 7). The results showed that the time factor would significantly reduce the BOP index, so that the longer the consumption time, the more the BOP index decreases. Intergroup changes in the BOP index did not cause a significant difference between the experimental and control groups (p = 0.205). A decrease of BOP up to 0.094 times in one week, 0.081 times in two weeks, and 0.0199 times in four weeks occurred after the intervention in the experimental group (Fig. 2).

**Table 6:** Comparison of the mean values of BOP, GI, PI, and OHI in patients with gingivitis referred to the private clinic

Variable	Test		Placebo		Independent T-test/ Mann–Whitney	Repeated measure/GEE
	Average	Standard deviation	Average	Standard deviation		
BOP-0	0.78	0.14	0.79	0.14	0.999	0.205
BOP-1	0.71	0.13	0.73	0.15	0.772	
BOP-2	0.62	0.12	0.71	0.15	0.076	
BOP-3	0.59	0.12	0.69	0.16	0.085	
Friedman-test	0.001		0.001			
GI-0	1.28	0.31	1.22	0.21	0.392	0.526
GI-1	1.13	0.31	1.10	0.21	0.811	
GI-2	0.95	0.25	1.05	0.26	0.372	
GI-3	0.86	0.23	1.04	0.22	0.006	
Friedman-test	0.001		0.001			
PI-0	1.14	0.22	0.94	0.15	0.002	0.315
PI-1	0.97	0.20	0.86	0.13	0.076	
PI-2	0.80	0.17	0.83	0.12	0.413	
PI-3	0.74	0.16	0.82	0.10	0.110	
Friedman-test	0.001		0.001			
OHI-0	1.88	0.25	1.66	0.14	0.001	0.078
OHI-1	1.71	0.27	1.55	0.13	0.007	
OHI-2	1.48	0.24	1.44	0.16	0.358	
OHI-3	1.41	0.21	1.42	0.15	0.489	
Friedman-test	0.001		0.001			

**Table 7:** Determining the effect of *Lactobacillus plantarum* MK06 probiotic suspension on BOP, GI, PI, and OHI indices using a generalized estimating equation model

The significance level	Degrees of freedom	Test statistics (Wald Chi-Square)	Source of changes	Parameter
0.001	1	103.514	Latitude of origin (Intercept)	BOP
0.205	1	1.605	Effect of group	
0.001	3	64.920	Effect of time	
0.001	3	25.456	Group-by-time interaction effect	
0.001	1	67.011	Latitude of origin (Intercept)	GI
0.526	1	0.401	Effect of group	
0.001	3	54.015	Effect of time	
0.001	3	27.069	Group-by-time interaction effect	
0.001	1	142.571	Latitude of origin (Intercept)	PI
0.315	1	1.012	Effect of group	
0.001	3	107.593	Effect of time	
0.001	3	60.749	Group-by-time interaction effect	
0.001	1	277.350	Latitude of origin (Intercept)	OHI
0.078	1	3.107	Effect of group	
0.001	3	135.939	Effect of time	
0.001	3	46.159	Group-by-time interaction effect	

#### Gingival index (GI)

The mean GI scores at baseline (GI-0) in the intervention and placebo groups were 1.28 and 1.22, respectively, but this difference was not statistically significant (p = 0.392). The mean GI at four weeks (GI-3) were 0.86 and 0.23 in the intervention and placebo groups, respectively, in which this difference was statistically significant (p = 0.006). According to the results of the Friedman test, during the study time, the changes in GI score had a statistically significant decreasing trend in both groups (p = 0.001) (Table 6). The percentage of GI changes was 32.81% in the intervention and 14.75% in the placebo groups, which may be clinically significant. However, there was no statistically significant difference between the two groups. Based on the generalized estimator equations model, considering that the significance of this test is less than 0.05, the time effect was significant (p = 0.001). In other words, the difference between the mean of the measurements in different time periods based on the Wald Chi-Square test was significant (Table 7). The results showed that the time factor could significantly reduce the GI index. Intergroup changes in GI index showed no significant difference between the experimental and control groups (p = 0.205). The interaction of time and intervention can significantly reduce GI (Table 7).

#### Plaque index (PI)

**Fig. 3:**
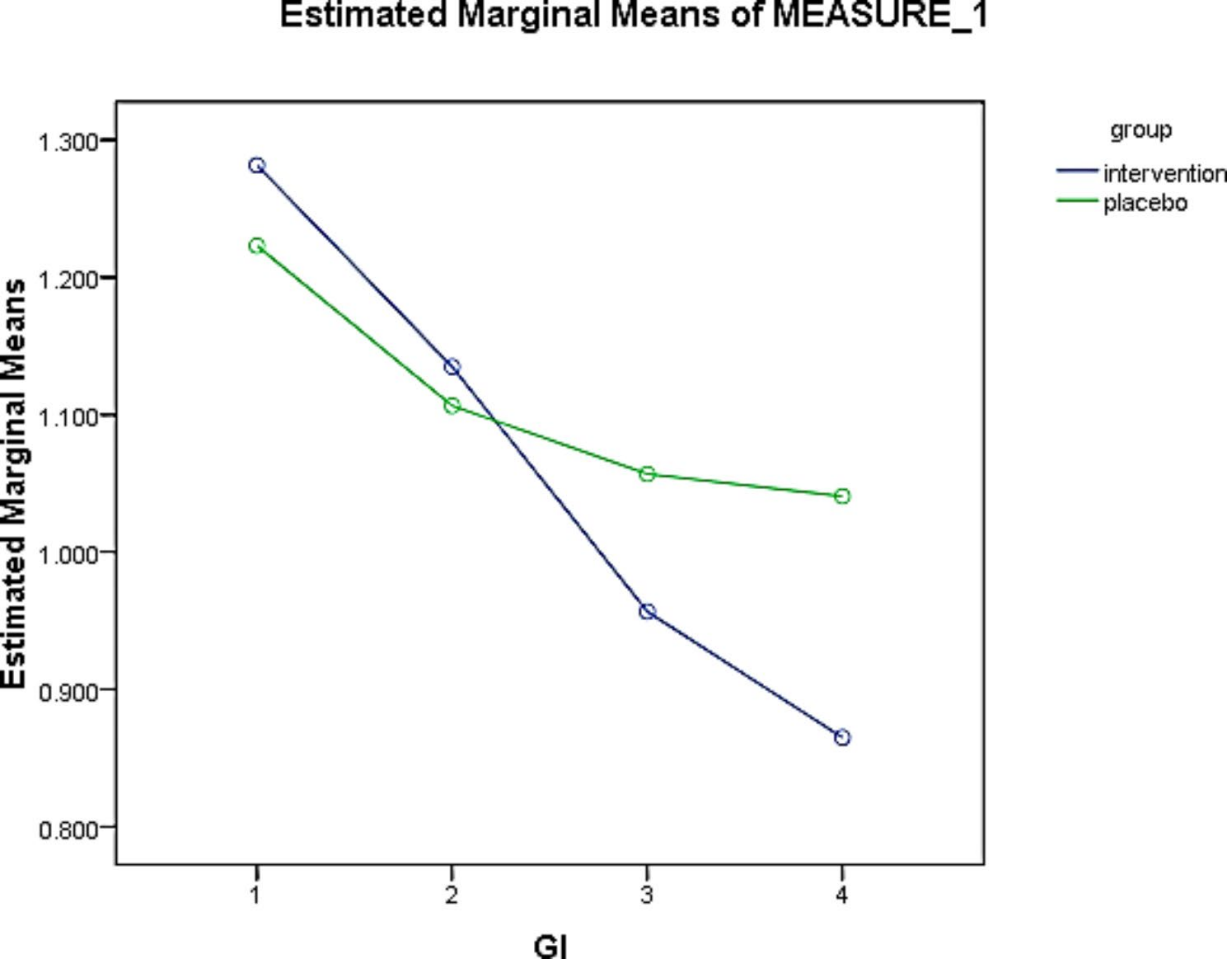
Comparison of the effect of *L. plantarum* probiotic suspension on mean GI values at 4 weeks

**Fig. 4:**
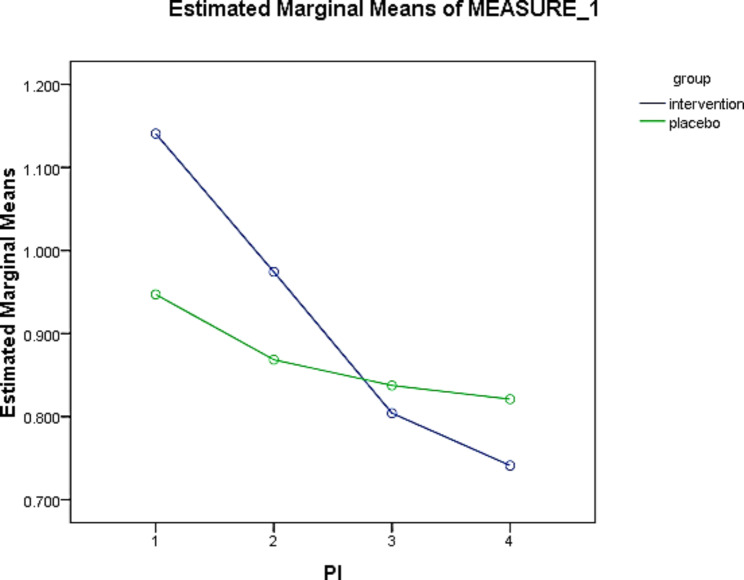
Comparison of the effect of *L. plantarum* probiotic suspension on mean PI values at 4 weeks

The interaction of time and intervention could significantly reduce GI. A decrease of 0.234 times in a week, 0.204 times in two weeks, and 0.076 times in four weeks occurred after the intervention in the experimental group (Fig. 3). The mean PI at four weeks (PI-3) were 0.74 and 0.82 in the intervention and placebo groups, respectively, in which this difference was not statistically significant (p = 0.110). According to the results of the Friedman test, during the study time, the changes in PI score had a statistically significant decreasing trend in both groups (p = 0.001) (Table 6). The percentage of PI changes was 35.08% in the intervention and 12.76% in the placebo groups, which may be clinically significant. However, there was no statistically significant difference between the two groups. Based on the generalized estimator equations model, considering that the significance of this test is less than 0.05, the time effect was significant (p = 0.001). In other words, the difference between the mean of the measurements in different time periods based on the Wald Chi-Square test was significant (Table 7). The results showed that the time factor could significantly reduce the PI index. Intergroup changes in the PI index showed no significant difference between the experimental and control groups (p = 0.315). The interaction of time and intervention can significantly reduce PI. The intervention in the experimental group over time resulted in a reduction of 0.472 times per week, 0.186 times in two weeks, and 0.047 in four weeks (Fig. 4).

#### Oral hygiene index (OHI)

**Fig. 5:**
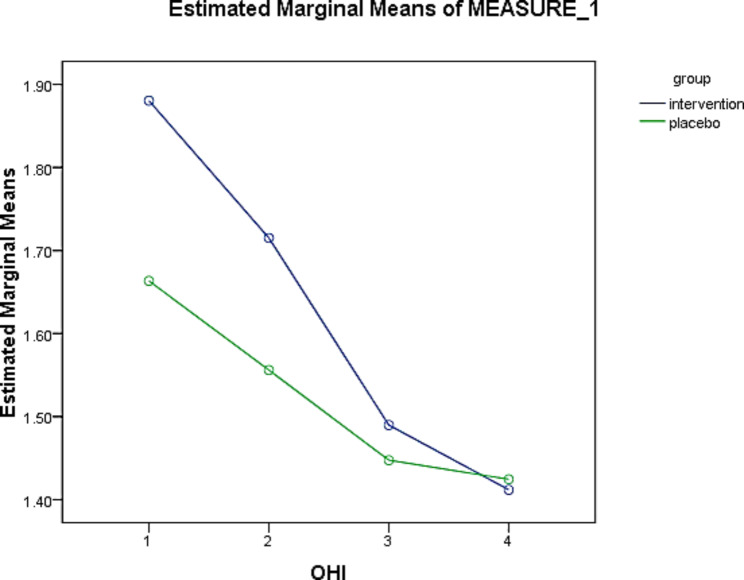
Comparison of the effect of *L. plantarum* probiotic suspension on mean OHI values at 4 weeks

The mean OHI scores at baseline (OHI-0) in the intervention and placebo groups were 1.88 and 1.66, respectively, and the difference was statistically significant (p = 0.001). The mean OHI at four weeks (OHI-3) were 1.41 and 1.42 in the intervention and placebo groups, respectively, in which this difference was not statistically significant (p = 0.489). According to the results of the Friedman test, during the study time, the changes in OHI scores had a statistically significant decreasing trend in both groups (p = 0.001) (Table 6). The percentage of OHI changes was 25% in the intervention and 14.45% in the placebo groups, which may be clinically significant. However, there was no statistically significant difference between the two groups. Based on the generalized estimator equations model, considering that the significance of this test is less than 0.05, the time effect was significant (p = 0.001). In other words, the difference between the mean of the measurements in different time periods based on the Wald Chi-Square test has become significant (Table 7). The results showed that the time factor could significantly reduce the OHI index. Intergroup changes in the OHI index showed no significant difference between the intervention and control groups (p = 0.078). The interaction of time and intervention could significantly reduce OHI (Table 7). The intervention performed in the experimental group over time led to a decrease of 0.230 times in a week, 0.172 times in two weeks, and 0.055 times in four weeks after the intervention (Fig. 5).

## Discussion

Periodontal disease and peri-implant mucositis/peri-implantitis are significant oral health challenges that can lead to tooth loss and implant failure. Conventional treatments like Scaling and Root Planing (SRP) and mechanical debridement have limitations, leaving a need for alternative approaches to reduce gingival inflammation and improve periodontal health. Probiotics have emerged as a promising option due to their ability to restore microbial balance, inhibit periodontal pathogens, and modulate the immune response. It highlights the potential of probiotics as a suitable alternative treatment to address gingival inflammation in both periodontal disease and peri-implantitis, suggesting their efficacy in improving clinical indices and microbiological parameters [4–6]. As we explore the benefits of probiotics in these contexts, the current study investigated the clinical effects of a native potential probiotic strain of *Lactobacillus plantarum* MK06 on gingivitis treatment. As anticipated, using *Lactobacillus plantarum* probiotic suspension served to lessen and treat those factors that trigger gingivitis. While the average scores of all indices BOP, GI, PI, and OHI decreased in both intervention and control groups over time, the rate of this reduction was longer in the experimental group than in the control group, and this difference was statistically significant. In fact, higher improvement was observed in the intervention group, which is clinically significant based on the percentage of parameter changes. Recruiting participants in this clinical trial was one of the most challenging tasks. The limitation in finding patients with gingivitis according to the inclusion and exclusion criteria caused the study to be conducted over a long period of time, preventing the study in the wider statistical population. Another limitation of this study was the uncertainty of the correct use of mouthwashes by patients, which tried to reduce its negative impact on the results by teaching proper use. Despite these limitations, however, unlike many previous studies conducted exclusively on children, the present study identified the effects of these probiotics on adults and different genders.

Reports of modest positive effects of probiotics in reducing clinical parameters associated with gingivitis suggest that more rigorous randomized clinical trials are needed to provide conclusive evidence on the efficacy of probiotics in gingivitis [20, 29]. Many studies have shown the effects of probiotics on clinical and microbiological parameters. These studies have focused on improving periodontal clinical indices and reducing pathogens belonging to the red and orange complexes in periodontal disease [6, 7]. Probiotic bacteria act by modulating the host’s immune system, directly competing with other microorganisms, or influencing microbial products. The specific effects of a well-characterized probiotic strain depend on its metabolism and surface molecules and produced bioactive compounds [30]. In a previous study, the inhibitory effect of different *Lactobacillus plantarum* strains against oral pathogens was examined. The highest inhibitory activity was observed for the *L. paracasei*, *L. plantarum*, *L. rhamnosus*, *L. casei*, and *L. salivarius* [31]. Although few reports are available on the inhibitory effect of *Lactobacillus plantarum* in the treatment of gingivitis, the use of different strains of *Lactobacillus plantarum* with probiotic potential for the treatment of periodontal disease has been increasingly considered in scientific circles related to oral diseases. Therefore, the present study was designed to use a native strain of *Lactobacillus plantarum* MK06 with potential probiotic properties to reduce important clinical parameters associated with gingivitis.

In a randomized clinical trial, the probiotic bacterial strains *Bacillus subtilis*, *Bacillus megaterium*, and *Bacillus pumilus* containing toothpaste, mouthwash, and toothbrush were used for eight weeks after the initial supra-gingival scaling [32]. It was stated that there is no statistically significant difference compared to placebo. In the present study, we used a different probiotic strain (*Lactobacillus plantarum* MK06) as a suspension without the patients undergoing scaling. The different results observed might be due to professional plaque removal, which may have subsided the effects of probiotics. In another study, after prophylaxis, patients received either a placebo or *Bifidobacterium* containing yogurt for twenty-eight days, and clinical measurements were recorded after five days of no brushing [33]. There was no difference between groups after the consumption of yogurts, but interestingly, significant improvement was observed in all clinical parameters after plaque accumulation. The results were in line with our study’s results. It is assumed that probiotic products can reduce the progression of the disease if the plaque is not professionally removed. In a previous study, the effect of probiotics and chlorhexidine-oral rinse on plaque buildup and gingival inflammation was examined [34]. The plaque index, gingival index, and oral hygiene index scores were recorded after oral prophylaxis at baseline, 14th, and 28th days. A significant difference was observed between both probiotic and chlorhexidine mouth rinse compared to the control. The results of this study were consistent with our results that *Lactobacillus plantarum* suspension can improve the clinical parameters associated with gingivitis. The results also suggest that probiotics could be a safe and effective alternative to chlorhexidine mouthwash. In another study, a randomized clinical trial was performed to ascertain whether patients having periodontitis benefit from adjunctive treatment with *Lactobacillus brevis* and *Lactobacillus plantarum* strains in the form of a gel as applied into periodontal pockets, thereafter taken as lozenges, to conventional scaling and root planing (SRP) [35]. The primary outcome measure was the number of remaining diseased sites with BOP and PD > 4 mm after three months. The results showed that probiotics were ineffective in reducing the number of persistent pockets [35]. In contrast, our study was performed on patients with gingivitis, and PD > 3 mm defined as exclusion criteria. Based on the promising results of our trial, it can be assumed that probiotics are more beneficial as a preventive measure in the early stages of periodontal diseases rather than a treatment for more advanced periodontitis. The results of this study show that the use of MK06 probiotic strain suspension has a significant effect in reducing the parameters related to gingivitis over time. The inhibitory ability, along with the lack of toxicity and resistance to probiotics, indicates that the probiotic suspension of *Lactobacillus plantarum* MK06 can be considered a promising adjunctive therapy for the treatment of gingivitis. However, more extensive clinical trials with further participants are required over an extended period owing to controversial outcomes between available studies. Furthermore, additional randomized clinical trials are required to corroborate the effects of probiotics on microbiological parameters.

## Conclusion

The study examined the clinical benefits of the probiotic strain *Lactobacillus plantarum* MK06 on the treatment of gingivitis and discovered that it significantly improved gingival indices compared to the control group. Challenges in participant recruitment and uncertainty about mouthwash usage were noted as study limitations. However, the study identified the beneficial effects of probiotics on adults with gingivitis and highlighted their potential as a safe alternative to chlorhexidine mouthwash. The results suggest that probiotics may be more effective as a preventive measure in early-stage periodontal diseases. Further rigorous clinical trials are needed to validate probiotics’ efficacy on microbiological parameters.

## Data Availability

The datasets generated during and/or analyzed during the current study are not publicly available due to minimal datasets that would be necessary to interpret but are available from the corresponding author on reasonable request.

## Data availability

The data sets analyzed during the present study are provided upon request from the corresponding author.
